# The introduction of new policies and strategies to reduce inequities and improve child health in Kenya: A country case study on progress in child survival, 2000-2013

**DOI:** 10.1371/journal.pone.0181777

**Published:** 2017-08-01

**Authors:** Marie A. Brault, Kenneth Ngure, Connie A. Haley, Stewart Kabaka, Kibet Sergon, Teshome Desta, Kasonde Mwinga, Sten H. Vermund, Aaron M. Kipp

**Affiliations:** 1 University of Connecticut, Department of Anthropology, Storrs, Connecticut, United States of America; 2 Jomo Kenyatta University of Agriculture and Technology, School of Public Health, Nairobi, Kenya; 3 Vanderbilt Institute for Global Health, Nashville, Tennessee, United States of America; 4 Department of Medicine, Vanderbilt University Medical Center, Nashville, Tennessee, United States of America; 5 Kenya Ministry of Health, Nairobi, Kenya; 6 World Health Organization/Kenya Country Office, Nairobi, Kenya; 7 WHO Inter-country Support Team for East and Southern Africa, Harare, Zimbabwe; 8 WHO Regional Office for Africa, Brazzaville, Congo; 9 Department of Pediatrics, Vanderbilt University Medical Center, Nashville, Tennessee, United States of America; The Hospital for Sick Children, CANADA

## Abstract

As of 2015, only 12 countries in the World Health Organization’s AFRO region had met Millennium Development Goal #4 (MDG#4) to reduce under-five mortality by two-thirds by 2015. Given the variability across the African region, a four-country study was undertaken to examine barriers and facilitators of child survival prior to 2015. Kenya was one of the countries selected for an in-depth case study due to its insufficient progress in reducing under-five mortality, with only a 28% reduction between 1990 and 2013. This paper presents indicators, national documents, and qualitative data describing the factors that have both facilitated and hindered Kenya’s efforts in reducing child mortality. Key barriers identified in the data were widespread socioeconomic and geographic inequities in access and utilization of maternal, neonatal, and child health (MNCH) care. To reduce these inequities, Kenya implemented three major policies/strategies during the study period: removal of user fees, the Kenya Essential Package for Health, and the Community Health Strategy. This paper uses qualitative data and a policy review to explore the early impacts of these efforts. The removal of user fees has been unevenly implemented as patients still face hidden expenses. The Kenya Essential Package for Health has enabled construction and/or expansion of healthcare facilities in many areas, but facilities struggle to provide Emergency Obstetric and Neonatal Care (EmONC), neonatal care, and many essential medicines and commodities. The Community Health Strategy appears to have had the most impact, improving referrals from the community and provision of immunizations, malaria prevention, and Prevention of Mother-to-Child Transmission of HIV. However, the Community Health Strategy is limited by resources and thus also unevenly implemented in many areas. Although insufficient progress was made pre-2015, with additional resources and further scale-up of new policies and strategies Kenya can make further progress in child survival.

## Introduction

**Fig 1:**
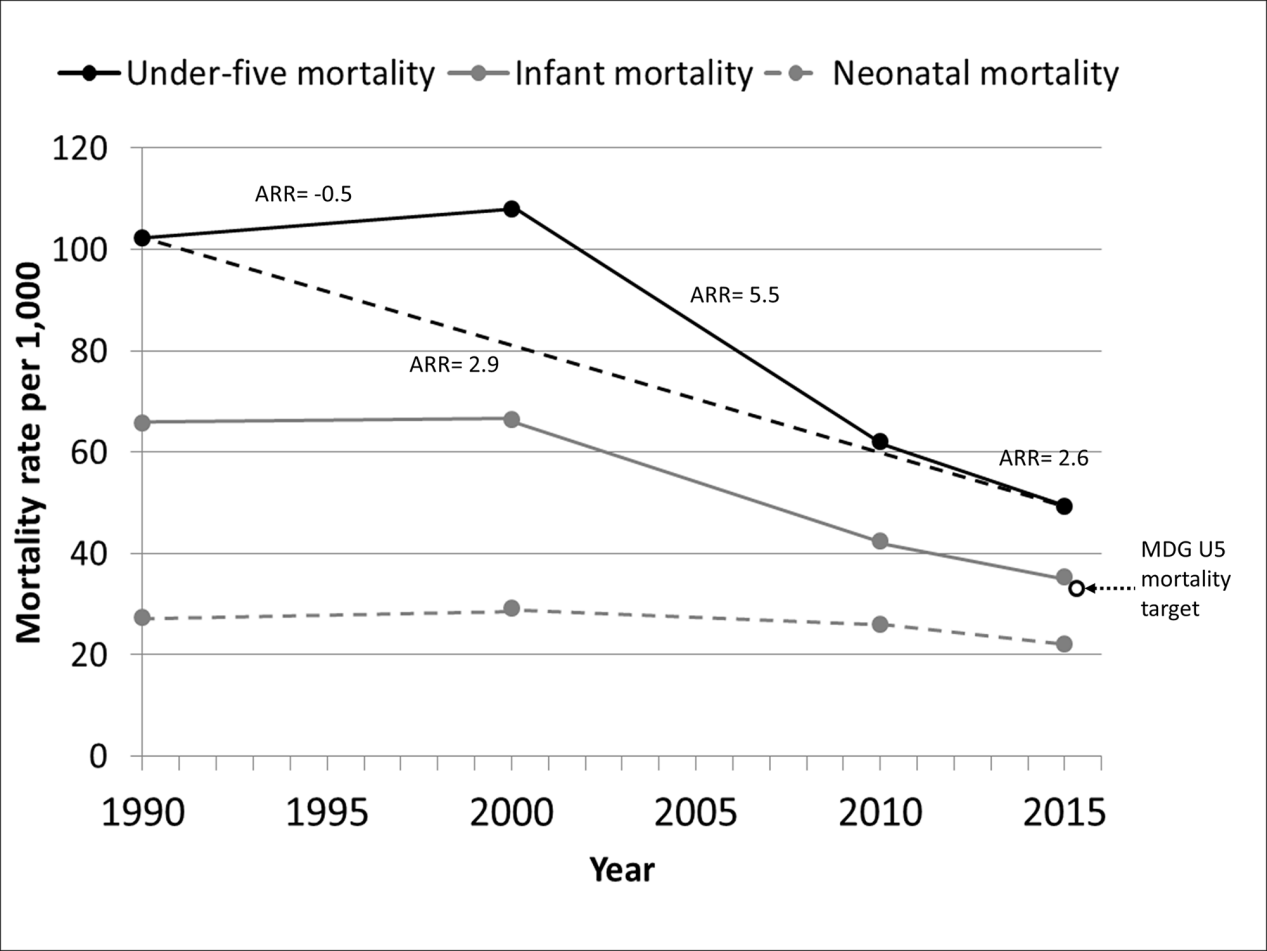
Under-five, infant, and neonatal mortality rates for Kenya in 1990, 2000, and 2013 (solid circles) with annual rates of reduction (ARR) for each period (solid and dashed lines) and the Millennium Development Goal (MDG) target (dotted arrow, open circle). Source: Levels and Trends in Child Mortality: Report 2015—Estimates Developed by the United Nations Inter-agency Group for Child Mortality Estimation [1].

Despite improvements over the last decade, sub-Saharan Africa continues to have the highest under-five mortality rates in the world at 83 deaths per 1,000 in 2015 [1]. The region as a whole did not meet Millennium Development Goal (MDG) #4 of reducing under-five mortality by two-thirds between 1990 and 2015 [2]. Nevertheless, as of 2015, 12 African countries had met their MDG4 goal [1]. There is, therefore, great interest in the reasons why some countries were able to meet MDG#4 while others were not. Kenya is a country that has not made sufficient progress in reducing under-five mortality. The under-five mortality rate in Kenya in 2015 was 49.4 deaths per 1,000 live births, down substantially from 107.9 deaths per 1,000 in 2000. However, this is only 52% lower than the 1990 rate of 102.3 per 1,000 due to an initial increase in mortality between 1990 and 2000. Infant mortality rates have also declined, though not as rapidly as total under-five mortality, while neonatal mortality has remained relatively unchanged (Fig 1).

In low and middle income countries such as Kenya, many unnecessary child deaths occur due to inadequate care during the prenatal, perinatal and early childhood periods despite the existence of numerous interventions that have been proven effective in preventing mortality among pregnant women and young children [3, 4]. In Kenya, although some studies have examined access and utilization of MNCH, most of these studies have been localized [5, 6] and focused on single interventions such antenatal care (ANC) attendance [7], immunization coverage [8, 9], delivery by a skilled birth attendant [7, 10] and community health services [5, 6, 11]. Fewer studies have explored the implementation and impact of recent national policies and strategies aiming to improve MNCH. Furthermore, most published evaluations have been quantitative [6, 12, 13]. Country-level case-studies assessing progress in reducing child mortality have been published for countries that successfully met MDG#4, including Niger, Uganda, Malawi, Ethiopia, Rwanda, Tanzania, and Zambia [14–19]. Until recently [20], there were no published case studies from countries with insufficient progress towards MDG#4 (note that the authors use the phrase “maternal, neonatal, and child health (MNCH)” in keeping with the language of international and national conventions. The paper, however, is focused on factors impacting MDG#4, which pertains only to the health of children under-five).

As part of a larger study on factors influencing progress in child survival in the Africa Region [19–21], Kenya was selected for an in-depth case study because of its low ARR in under-five mortality. The objective of the parent study was to assess barriers and facilitators within and across countries either on-track or not on-track to meet MDG4. Comparisons across countries will be reported elsewhere. Here, we evaluated national policy and strategy documents, quantitative indicator data, and qualitative data to identify barriers and facilitators influencing child survival in Kenya. We report on the overarching factors contributing to Kenya’s progress in reducing under-five mortality that emerged from the case study: widespread socioeconomic and geographic inequities (including location in urban “slum” communities or rural areas) to access and utilization of MNCH services and national efforts to reduce these inequities.

## Methods

The period of interest for this case study and the parent study on child survival in Africa was 2000–2013. Indicator data were obtained for years closest to 2000 and 2013 (details below), and national policies and strategies issued between 2000 and 2013 were also obtained. Key informant interviews and focus groups with community women occurred in 2013. The national document review, key informant interviews, and focus group discussions sought to explore the following eight content areas based on global strategies [22–27] related to child survival: 1) Health care system (including leadership, structure, human resources for health, access & utilization, monitoring & evaluation, and accountability), 2) National health strategies and policies (and regulations and laws, when applicable), 3) MNCH interventions, 4) Clinical standards and guidelines, 5) Commodities and essential medicines, 6) Financial flows and resources, 7) Effective partnerships, and 8) Other contextual factors (e.g., conflict, political environment, hygiene and sanitation, nutrition and food security, education, and human rights). Focus group discussions with community women focused only on the health care system, MNCH interventions, medicines, and contextual factors.

### MNCH indicator data

Data were obtained on the core indicators monitored by Countdown to 2015. Most data were obtained from the World Bank Data Catalogue [28], a repository of national, regional, and global indicator data compiled from officially-recognized, international sources, including national Demographic and Health Surveys (DHS) and other surveys. While specific data sources differed from one indicator to another in the Data Catalogue, data sources and methods were consistent over time for any given indicator (see Kipp, et al. [21] for additional detail). Data for indicators not readily available from the World Bank Data Catalogue were obtained directly from the 1998 and 2014 Kenya DHS [29, 30].

Given the scope of the larger study within which this case study is nested and recognizing that indicator data are not always available for the exact years of interest, this study included those data that most closely corresponded to the beginning of the study period in 2000 (range 1998–2003) and end of the study period in 2013 (range 2009–2014). This enabled us to document the net change in coverage of key MNCH indicators by the end of the study period, relative to the beginning.

### Review of MNCH policies and strategies

An information abstraction guide was developed based on the general content areas listed above and the cross-cutting questions in Table 1. Policies and strategies pertaining to overall national health, MNCH, and those from other sectors related to MNCH (e.g., education, water and sanitation, and agriculture and nutrition) were obtained from the WHO African Region office, the WHO country focal points for Kenya, and Kenya’s Ministry of Health (MOH). These documents were reviewed and any additional documents referenced and deemed important were obtained from WHO or MOH. The final list of reviewed documents can be found in S1 Table.

**Table 1:** Key questions and themes explored during the review of national health policies and strategies, key informant interviews, and focus groups with community women that cut across child survival content areas.

Specific questions for review of national policies and strategies	Specific themes explored across content areas with key informants	Specific themes explored across content areas with community women
What policies and strategies related to child health were in place between 2000 and 2013 (including changes during this period)?	Issues related to program evaluation, access and utilization, coverage, impact, and sustainability, as appropriate.	Barriers and facilitators to accessing and utilizing MNCH services, including cultural and community factors.
What challenges were stated as hindering progress towards MDG#4?	Knowledge and experiences related to MNCH across the *health care continuum* (prenatal care through age 5 years).	Experiences related to MNCH across the *health care continuum*.
What facilitators were stated as enabling progress towards MDG#4?	Knowledge and experiences related to MNCH across the *health system continuum* (community to tertiary hospitals).	Experiences related to MNCH across the *health system continuum*.
What plans for change or improvements were either implemented after 2013 or were proposed as a measure to improve child survival?		

Each document was reviewed multiple times by one author (CAH) and a second reviewer (MAB) was consulted if further clarification on the document findings was needed. Information was recorded as outlined in the abstraction guide to standardize abstraction and summarization of content across documents. In order to avoid biased interpretation of the information documented, the abstracted information was reported as it was stated in the original source and efforts were made to avoid overstating or minimizing the original information or adding commentary not contained in the source.

### Qualitative procedures

#### Study location and participants

Because important differences in MNCH often exist between urban and rural areas, participants for the qualitative study were included from both urban and rural areas. Nairobi (Nairobi Province) was selected as the urban location and the areas surrounding Embu (Eastern Province) were chosen as a representative rural location. Both Nairobi and Eastern Provinces had under-five mortality ARRs comparable to the national ARR based on Kenya DHS data from 1993 [31] and 2008/09 [32].

Data were obtained from semi-structured, key informant interviews with Kenya MOH officials, donor partners, community-based organizations (CBO) involved in MNCH, and health care providers (HCP). Data were also obtained from four focus group discussions (two in Nairobi, two in Eastern Province) with 40 women who have experience accessing MNCH services for at least one child. Interviews and focus groups were conducted between October 6 and December 6, 2013.

#### Eligibility criteria and identification of study participants

All participants, whether key informants or focus group women, were eligible for the study if they met the following criteria: 1) 18 years of age or older, 2) having adequate knowledge or experiences related to childhood survival specified for each participant group below, 3) English- or Swahili-speaking, and 4) being able to provide written or verbal informed consent. Specific inclusion criteria for each key informant group included the following: national or provincial-level officials working in government-level health care system administration, policy-making, program development, leadership, or any aspect of MNCH (MCDMCH officials); directors, managers, or other leaders of entities providing financial or other aid for MNCH services, or international or national organizations focusing on MNCH or with MNCH as one component of their mission (Donor organizations); directors, leaders, or managers working for a CBO involved in or providing referrals to MNCH services; and professionally trained physicians, nurses, clinical officers, or other health-related staff working in a health facility providing MNCH care (HCPs). For the FGDs, women with children (either living or deceased) aged five years old or younger who had sought MNCH services within the previous twelve months were purposefully sampled.

Similar numbers of participants from each key informant group were enrolled, and a range of ages, work experiences, and positions/roles within each group was sought. Additionally, efforts were made to balance the number of urban and rural participants among the HCPs and CBO workers. Lists of potential key informants from each group were developed by the in-country research team with assistance, as needed, from the WHO focal points and an MOH official involved with Child and Adolescent Health. A letter signed by an official from the MOH was sent to each potential key informant participant informing them of the purpose of the study, risks and benefits of participation, and describing the interview process. These were followed-up with a phone call or email from the research team to set up a meeting time for those interested. Basic demographic characteristics of the key informants are shown in Table 2.

**Table 2:** Characteristics of key informants in Kenya.

	Ministry of Health (N = 9)	Donors* (N = 8)	Community-based Organizations** (N = 13)	Providers† (N = 13)
**Sex, N (%)**				
Male	7 (78)	5 (62.5)	6 (46)	5 (38)
Female	2 (22)	3 (37.5)	7 (54)	8 (62)
**Age**, **Median (IQR)**	50 (45, 52)	49 (40, 55)	40 (39, 43)	48 (41, 50)
**Ethnicity, N (%)**				
Embu	1 (11)	1 (12.5)	5 (38)	2 (15)
Kalenjin	1 (11)	1 (12.5)	0 (0)	0 (0)
Kamba	1 (11)	0 (0)	0 (0)	2 (15)
Kikuyu	1 (11)	2 (25.0)	4 (31)	2 (15)
Kisii	1 (11)	1 (12.5)	0 (0)	0 (0)
Luhya	1 (11)	1 (12.5)	3 (23)	0 (0)
Luo	2 (22)	1 (12.5)	0 (0)	1 (8)
Meru	0 (0)	0 (0)	0 (0)	3 (23)
Other††	1 (11)	1 (12.5)	1 (8)	3 (23)
**Education, N (%)**				
Secondary	0 (0)	0 (0)	6 (46)	0 (0)
Post-secondary	8 (89)	8 (100)	7 (54)	13 (100)
Missing	1 (11)	0 (0)	0 (0)	0 (0)
**Years working for organization, Median (IQR)**	23 (20, 26)	10 (6, 12)	6 (5, 9)	10 (4, 17)

*4 from international donor organizations, 4 from national organizations.

**6 CBO participants from Nairobi (urban site), 2 from Embu; includes faith-based (3), private (3), and others with unstated affiliations (7).

^†^7 from Nairobi (urban site), 6 from Embu; includes public/government hospital or clinic (11) and private, non-faith-based hospital (2).

^††^ Other includes one each of Mijikenda and Taita, or not stated (n = 4)

Women were recruited to participate in focus groups using snowball sampling. The research assistants also visited healthcare facilities to advertise the study. The two urban focus groups were held in Nairobi, one at a local hospital and one at a community center. The two rural focus groups were held at Embu County Hospital. Women were recruited primarily from the rural areas of Embu County; the hospital served as a familiar and central meeting location. For all focus groups, a multi-pronged approach was used for recruitment, including: a list of eligible women developed by community volunteers; snowball sampling in which eligible women were asked to refer their peers with children (living or deceased) aged five years old or younger; and advertising through the health facility. We did not ask healthcare providers or MoH officials for referrals to avoid bias. The FGDs were conducted by experienced social scientists who were aware of the possible effects the venue might have on interview dynamics, and the interviewers attempted to minimize this effect by conducting the FGDs outside of the hospital buildings. As with the key informants, a balance was sought in the level of education and the participants with live and deceased children, as well as a diversity of experiences and opinions regarding MNCH. All FGD participants were reimbursed for their time and travel at a maximum cost of 500 Kenyan Shillings (approximately 5 USD). Written informed consent was obtained from all enrolled participants. Basic demographic and health characteristics of the community women are shown in Table 3.

**Table 3:** Characteristics of female focus group participants in Kenya.

	Rural participants (N = 18)	Urban participants (N = 22)
**Age**, **M (IQR)**	25 (24, 27)	30 (24, 38)
**Ethnicity, N (%)**		
Embu	8 (50)	0 (0)
Kamba	1 (6)	1 (5)
Kikuyu	6 (38)	8 (40)
Kisii	0 (0)	2 (10)
Luo	1 (6)	5 (25)
Meru	0 (0)	2 (10)
Other*	0 (0)	2 (10)
**Education, N (%)**		
None	--	1 (5)
Primary	5 (29)	9 (41)
Secondary	10 (59)	8 (36)
Post-secondary	2 (12)	4 (18)
**Travel time to health care (dry season), N (%)**		
Less than one hour	9 (50)	9 (41)
One to two hours	7 (39)	10 (45)
More than two hours	2 (11)	3 (14)
**Number of living children, M (IQR)**	1 (1, 2)	2 (2, 2)
**Age of youngest child, M (IQR)**	1 yr (5 mo, 2 yr)	1 yr (7 mo, 4 yr)
**Any children who died <5yrs old, N (%)**		
No	14 (82)	18 (82)
Yes	3 (18)	4 (18)
**Place of delivery for latest pregnancy, N (%)**		
Health facility	18 (100)	19 (86)
Home	0 (0)	3 (14)
**Birth Attendant for latest pregnancy, N (%)**		
Doctor	12 (67)	10 (45)
Nurse/midwife	5 (28)	9 (41)
Other health worker	0 (0)	1 (5)
Traditional birth attendant	1 (6)	2 (9)

* Other includes one Msukuma and one Arab

#### Interview and discussion guides

Interview guides for key informants and discussion guides for focus groups with community women were developed, pilot tested through cognitive interviewing [33], and revised as needed. The guides focused on barriers and facilitators for improving child survival in areas related to MNCH, corresponding to the general content areas described above for the review of national health policies and strategies and the cross-cutting questions in Table 1. Not all topics were appropriate for each key informant group, but each topic was asked of at least two of the four groups. While participants were encouraged to discuss the entire period from 2000 forward, most participants recalled more recent information and experiences.

#### Data collection and analysis

Key informant interviews were conducted in English by one research assistant using the appropriate interview guide and were audio recorded. The focus groups were in Swahili and also audio recorded. Two research assistants were present at each focus group to facilitate discussion and note-taking. Audio recordings were transcribed by the research assistants, translated into English as needed, and field notes incorporated into the transcript. Transcripts were coded and analyzed using the qualitative software Atlas.ti [34]. Deductive themes were determined *a priori* based on interview guides and key topics of interest based on literature review. Additional themes were also identified upon review of the transcripts. Text was coded and reviewed for patterns of consistency, variation, relationships between themes and exemplary cases or quotations [35, 36]. Ethical approval for the qualitative portion of the study was obtained from the Kenyatta National Hospital/University of Nairobi Ethics and Research Council and from Vanderbilt University Medical Center Institutional Review Board.

## Results

### MNCH coverage indicators

**Fig 2:**
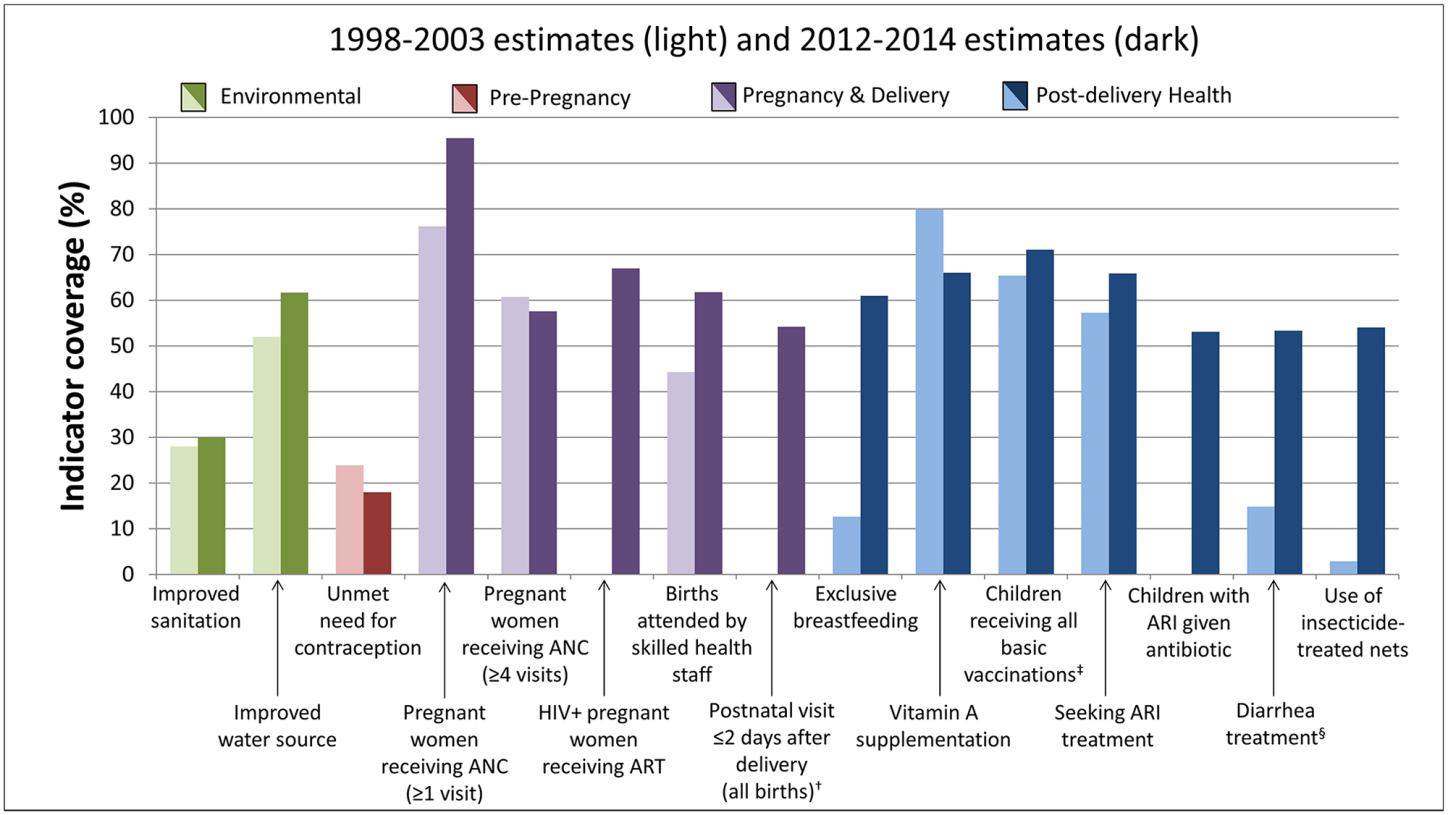
Changes in child survival indicator coverage in Kenya, 2000 and 2013*. *Estimates were not always available for years 2000 and 2013, in which case the nearest estimate between 1998 and 2003 or 2012 and 2014 was used; **data were not available for the three indicators showing no coverage during the 2000 time period**. ^†^Among all births, both inside and outside a health facility ^‡^ Children 12–23 months old who have received Bacillus Calmette-Guérin, measles and three doses each of diphtheria, pertussis, and tetanus and polio vaccine (excluding polio vaccine given at birth) ^§^Children under 5 receiving oral rehydration and continued feeding Source: Kenya DHS [29, 30] and the World Development Indicators Data Catalogue from the World Bank [28] (accessed August 2015)

Kenya has improved coverage of 10 of the 15 core indicators shown in Fig 2 for which data were available for both 2000 and 2013. Data from the 2000 time point were not available for HIV-positive pregnant women receiving antiretroviral therapy (ART), postnatal visit, and children with acute respiratory infection (ARI) given antibiotics. Highest current indicator coverage was for women receiving at least one ANC visit (96%), meeting a need for contraception (82%), children receiving all basic immunizations (71%), and HIV-infected pregnant women receiving ART for prevention of mother-to-child transmission of HIV (PMTCT) (67%). Coverage was below 50% for improved sanitation (30%) only. Declines during the study period only occurred for pregnant women receiving at least four ANC visits and Vitamin A supplementation.

### National document review and qualitative study

#### Description of inequities in MNCH

Inequities were nearly universally described in national documents and qualitative data as a major barrier to child survival in Kenya. National documents described inequities as occurring along geographic lines (with urban dwellers having generally greater access and uptake than those in rural areas), socioeconomic status (with impoverished populations in both urban and rural settings having poorer access and utilization), and gender (pronounced gender inequities affecting women’s abilities to obtain necessary care for themselves and their children) [37, 38]. As exemplified in the quotations below, key informants and community women described inequities in much the same way, while also identifying additional inequities based on tribal/ethnic affiliation and a provider’s perception of a patient’s social standing. Some participants further suggested that those from particularly marginalized groups had greater difficulty accessing care and would experience harsher treatment from providers, preventing them from returning for additional care.

“If you are staying in areas such as Garissa or where security, infrastructure, water is an issue and therefore human resource, there are very few human resource working there, not motivated enough to stay there, they are posted there, they work and then they leave then accessibility will depend on the residence. Whereas if you are staying in Nairobi where you have everything, all the hospitals including the referral, the specialists then you have the best treatment. But also it also depends on if you are in Mathare in Nairobi or Kibera in Nairobi [Mathare and Kibera are slums in Nairobi] then poverty will also lead you to the public hospitals where you have limited care.”

(44 year old, female HCP)

“What I would say is that in government hospitals, one can easily jump the queue especially if you happen to know anyone who works there; but a person like me who does not know anyone gets to stay on the queue for long. So I think they should treat all people as the same.”

(38 year old urban woman with 3 children)

The national document review and qualitative data highlighted three specific national-level strategies that were implemented during the study period to address inequities and expand equal access to specific MNCH services: removal of user fees, the Kenya Essential Package for Health (KEPH), and the Community Health Strategy. While these were not the only policy efforts implemented during the study period, these were the efforts most commonly discussed by participants. Kenya’s policy to exempt user fees for maternal and child (under-five) healthcare was instituted in 2004 as a measure to remove financial barriers to care from public dispensaries and health centers. This policy was also known as the “10/20 policy”, referring to the flat registration fee of ten Kenyan shillings (KES) and 20 KES charged at dispensaries and health centers. In 2007, the Kenya Essential Package for Health (KEPH) and the Community Health Strategy were established under the Second National Health Sector Strategic Plan [39]. The KEPH defined service provision across a continuum of care including six service delivery levels and six age cohorts, specifying a defined set of interventions and services for each point of care. By providing integrated packages of care and interventions at each level of the health care system, the KEPH also aimed to improve the referral system to ensure that patients first access care at the lower primary care level and are only referred to specialty care at higher levels as needed, rather than patients seeking initial care at overburdened tertiary centers, as was reported by several key informants. As one healthcare provider explained, “What we need is to make sure that we have enough health workers in the lower levels so that we shall have fewer patients who are coming to the referral hospitals. Let only the patients who need the care of the specialist are the ones who are coming to the referral hospital.” The KEPH also standardized the types of medicines and other health inputs that should be available at each level of the system, and put into place a system for ensuring a more reliable flow of essential medicines and commodities into facilities. To support and implement the KEPH in under-served areas (especially rural areas), plans were also made to upgrade and expand healthcare facilities.

The National Community Health Strategy was also established in 2007 to bring services to the local level, strengthen treatment and preventive interventions at the family and community levels, and facilitate referrals from the community to appropriate healthcare providers. The Community Health Strategy also emphasized the use of community health workers (CHWs) to communicate health information, promote disease prevention and management, improve nutrition and increase use of water, sanitation and hygiene practices. This Strategy aimed to increase awareness and use of community services through advocacy, mass media campaigns, and presentations to religious and other community-based groups and provincial/district administrations. To accomplish these activities, CHWs have been trained in health education, making appropriate referrals, measures to ensure follow-up and continuity of care, and even in the treatment of some basic childhood illnesses at the community level. In addition, the strategy also established the development of “community units” that are linked to health facilities and use Community Health Extension Workers (CHEWs) to assist with coordination of referrals and follow-up between the community and higher health system levels.

#### Implementation of strategies and programs to reduce inequities in MNCH

Qualitative data provided further understanding of the implementation and impact of the policies described in national documents. Regarding the impact of removing MNCH user fees, both key informants and community women described free care as a major strength of the healthcare system that improved access to and utilization of MNCH services by enabling low-income families to more easily obtain care. However, they also reported implementation of this policy has been uneven. Both key informants and community women noted that even when there is no charge for the health care visit, patients still face “hidden” out-of-pocket expenses such as the costs of health cards or booklets, transportation to health facilities, and medications when stock-outs at public facilities force families to purchase medicines from private pharmacies. In addition, key informants, as well as national documents, noted that free services have increased demand and created an additional strain on already over-burdened facilities, particularly at the primary care level and in underserved areas, thereby perpetuating inequities.

“…the ANC [antenatal care] profile at least is being done free. For the child, the services are free, so at least the government is spending money to make sure that these services are being given. Then also the government has given free maternity delivery services, which contribute to mothers coming to deliver in the hospital without paying anything, but maybe the services will be taken up slowly. It is also a government policy that all people should go to hospital; especially under-fives are the responsibility of the government.”

(54 year old, female CBO partner)

“Although antenatal we say it is free, there is the other cost to the mother of being away from home, bus fare, needing fare from another person if herself she doesn’t have an income.”

(48 year old, female, Ministry of Health official)

“For people who may not have enough money; especially those who are not working, it becomes a problem because they need money to pay hospital bills and also for transport. Again we are usually told to buy medicines even after visiting the hospitals.”

(26 year old, urban woman with 3 children)

Although the KEPH was added to the Kenyan healthcare system later in the study period, both key informants and community women noted that access to MNCH services was increasing as a result of newly constructed and upgraded healthcare facilities in underserved areas and expanded service provision as illustrated in the quotations below.

“What I’m saying…is [that] these are the very front line first services that are provided at any level in the healthcare system starting from level one all the way to level six as we know it in the KEPH document. And that’s why the first major service to be at a lower level is the maternity service… Why? It is because of the importance, when we know that mothers should not be referred when they come from home to deliver in a health facility…So maternal and newborn and child health services, I would say they are the front line services in any health facility and therefore where we have a health facility, these services are there in Kenya.”

(53 year old, male, MOH official)

“Building of dispensaries has really helped because there are those who are far in the bush, there are dispensaries nearer or there are doctors who go there. So if the child needs a certain vaccine you will take her there instead of taking her to a far off facility where one would require some money for transport…so it has brought healthcare closer to the people in the villages…This way the people are able to access say, the immunization without having to use transport to come to such a big hospital.”

(25 year old, rural woman with 1 child)

However, key informants and national documents suggest that many facilities have not been upgraded to provide EmONC and neonatal care as intended through the KEPH. The uneven availability of EmONC and neonatal care may perpetuate uneven access to services. In addition, community women reported that distance to facilities and lack of money continued to be major barriers to accessing other MNCH interventions, particularly for pregnant women and infants, further emphasizing incomplete implementation of both the KEPH and policies for free care.

“…what has not been implemented, I would say like if you look at the emergency in maternal and newborn care, there’s a package that was meant to be rolled out in most of the hospitals. Not all hospitals really got that or even the health providers know about that package. And as such we know that not all hospitals in our country are implementing that. One maybe lack of knowledge or secondary maybe whoever implemented never followed it up. So that’s another area I’ve seen that much as we have tools much as we have guidelines, there are still not fully implemented or rather they’re still not fully taken up by the healthcare providers. And postnatally the follow up at MCH we still have a lot of challenge also on the way the mothers are taking up those services.”

(40 year old, female HCP)

**“**When you look at Kasarani [near Nairobi], yes it is a government hospital, and a woman may be going there for antenatal clinics but the maternity is not there. When it comes the time to deliver she will have to go very far. You may find that a woman ends up giving birth on the way because the maternity is too far from where she lives.”

(35 year old, urban woman with 6 children)

The KEPH was also intended to improve availability of essential medicines and commodities, but many key informants at multiple levels of the system indicated that this had not been accomplished by the end of the study period. They further stated that due to lack of training, facilities do not always make medicine and commodity requests in a timely manner, preventing facilities from offering the services and medicines required by the KEPH. Stock-outs of essential medicines and commodities were common across facilities, but were described by key informants as most acute at primary care facilities. Poor availability of essential medicines and commodities was also reported as a major barrier to care by community women.

“…we’ve had incidents where we’ve actually been missing key drugs that are necessary… What now the facilities are doing is that…they’ve been drawing from an allocation that they have and I think one of the things is that they would still need to be trained on really making requisition of some of these essentials…because you know they would just require what they think that they easily…consume and prominence might not be given to some of the essential medicine that are required for the survival of the child and mother. So we would need even a bit of re-training of people on requisition of medicines that they would want…”

(43 year old, male MOH official)

“They should try and facilitate us with that medicine so that we don’t go and buy them at the chemist, because in the chemist they are expensive, and here we can get them for free.”

(25 year old, rural woman with 1 child)

During the interviews and focus group discussions, there was much discussion of community level MNCH care, related to the implementation of Kenya’s National Community Strategy. Nearly all key informants highlighted this Strategy as an effective component of the MNCH system. They described two cadres of community-based health care workers expanded through the Community Strategy, CHWs and CHEWs, as valued facilitators of child survival through their promotion of MNCH care utilization. CHWs and CHEWs were also described as improving continuity of care by strengthening the health referral system from the community to the facility level, as illustrated in the quotations below.

“At least at the community level, we have community health workers who are being trained on prevention at the household level. So at least that one is working well and then mothers, mothers at least can access information from those health workers if they are interested. Then there are also programs which targets community level to give them information. I think that is working well.”

(54 year old, female CBO partner)

“I can say those services are there, because in the communities where we come from, there are people who are able. For example, this one is a private hospital [*referring to hospital where interview was conducted*] and if you bring a child here, they will be treated. But if a community health worker finds that a person can’t afford this place, they either refer them to Kasarani or Kiambu *[Government owned facilities*] and when they go there, they will get treated and come back healthy.”

(42 year old, urban woman with 5 children)

While Kenya’s Community Health Strategy was largely felt to be effective, several key informants expressed concern that not every facility has been linked with a community unit and that not all community units are yet fully functional in terms of adequate staffing or supplies. Furthermore, a few key informants stated that the only fully functional community units were those supported by external partners, whereas those supported by the government were less effective. Community women expressed appreciation of the care and education provided by CHWs, but recommended that the government train and pay more community members as CHWs as a means of increasing health education in the community.

“I want to add that the government should train more community health workers especially from the villages; So that if someone reports to the chief about a woman who is sick, the chief shouldn’t send them away but would listen and find means of helping that woman and also reach other women in the villages.”

(42 year old, urban woman with 4 children)

“The community strategies and home visits are working in a few select places, not in all centres and therefore I cannot say that it works uniformly well in….the whole country. In some places it works quite well where it is supported by private partners, the non-governmental organizations, and other well-wishers. But where it’s fully government funded, it is not working very well.”

(37 year old, male donor partner)

“Yes, because I have said not all units or sub-locations have community health workers and also those who do have community health workers, they are not fully equipped with skills. So I think there on that side it’s a bit poor.”

(40 year old, male CBO partner)

The qualitative data also indicate that the National Community Strategy has positively impacted intervention coverage. Efforts at the community level, although incomplete, were attributed to successes by both key informants and focus group participants in the implementation and coverage of immunizations, malaria prevention, and PMTCT services. Key informants and community women attributed success of these interventions to long-term educational and awareness campaigns and expanded availability of services through mobile clinics and local primary care dispensaries.

“…the quality is good because there are these community health workers who educate the women in the community, so when they go to the clinic they are taught how to take care of the baby.”

(40 year old, urban woman with 2 children)

“It is especially through integration of services and making those interventions available in the lower level facilities, you know dispensaries. And even outreach for things like immunization, Vitamin A, and, once in a while, campaigns to try and bridge the gap.”

(40 year old, female MOH official)

“I think PMTCT has done so well because it has gone up to the dispensary level, even at the community, mothers are told when they are pregnant they can go for testing.”

(54 year old, female CBO partner)

## Discussion

This case study evaluated Kenya’s national policies and strategies, qualitative data, and quantitative indicator data related to child health. Under-five mortality in Kenya decreased 52% between 1990 and 2015, which was not sufficient to attain MDG#4. During this time, coverage of core MNCH indicators also increased substantially, with few exceptions. The removal of user fees, the Kenya Essential Package for Health, and the Community Health Strategy likely contributed to the substantial increase in these indicators. However, the case study also highlights the role of persistent inequities in healthcare access and service delivery that have impeded progress in reducing under-five mortality. The implementation and impact of efforts made to reduce these inequities through the removal of user fees, the Kenya Essential Package for Health, and the Community Health Strategy were not complete by the end of the study period. However, they nonetheless indicate a focused commitment by Kenya to expand the equitable availability of MNCH services and accelerate progress in reducing under-five mortality.

The global picture of inequities, where MNCH intervention coverage and child survival are worse in remote areas, impoverished and marginalized households, and for mothers with limited education [40–42], is mirrored in Kenya, with inequities largely due to socioeconomic status and location in urban “slum” communities or rural areas which ultimately affect access and utilization of MNCH services [9, 13, 43–46]. While the presence of inequities in child health is therefore not unique to Kenya, we found the amount of attention and discussion pertaining to inequities to be remarkable, as this was not a major theme identified in our other country case studies [19, 20]. Not surprisingly, the impact of addressing inequities on child survival has been described, documenting that countries achieving the most notable gains were able to increase coverage among the poorest populations at a faster rate than for the wealthiest [40, 47]. This demonstrates that investing in the health of the most marginalized populations can drastically improve child survival and have widespread impacts on a country’s economy [24], an approach that Kenya has pursued.

User fees had previously hindered utilization of MNCH services in Kenya and the 2004 policy to provide free care has indeed improved access to MNCH services in many areas, consistent with other studies conducted around the world [15, 41, 48–53]. However, in Kenya as in other resource-limited settings, inequities remain due to other barriers including out-of-pocket costs that include lost wages, travel, food and accommodations during hospitalization or care of a sick child [54, 55]. These costs keep essential MNCH care completely out of reach for the poorest and most vulnerable [55–57]. Furthermore, while removing user fees does increase uptake, implementation in Kenya has been uneven as facilities lack the resources to fully adhere to the policy, particularly in rural, under-resourced, or marginalized areas. Increased patient volume due to user fee removal may further strain resources at the primary care level, such that careful planning management and support from other policy measures must be ensured so that quality of care is maintained [55, 58–60]. If these challenges can be adequately managed, national policies to reduce or eliminate out-of-pocket costs can successfully alleviate inequities hindering access to needed MNCH services in resource-limited settings as has already been demonstrated in other countries in Sub-Saharan Africa [50, 51, 61].

A second important strategy working to provide equal access and utilization of MNCH services is the provision of integrated packages of services across all life stages and at each level of the health system, as exemplified by Kenya’s KEPH [56]. Qualitative data from both key informants and community women suggests success and ongoing challenges in delivery of the KEPH. One important component of the KEPH specifically aimed to improve access and reduce inequities in maternal and child outcomes. Community women, in particular, reported increased availability of facilities. However, implementation of the KEPH has been slow, such that many facilities across levels of care have not yet been upgraded to fully provide important maternal and child services, including critical EmONC and neonatal care. Frequent stock-outs of essential medicines and commodities, particularly at the primary care level, have further minimized the impact of the KEPH, especially in marginalized areas. The delivery of essential packages of care across the reproductive and MNCH continuum of care is now recommended by the WHO [62], and many of the challenges Kenya faces, including frequent stock-outs, have been reported in other countries [63–67]. These challenges, combined with increased demand due to removal of user fees and increased engagement at the primary care level, demonstrate the need to continually invest in multi-pronged efforts to provide equitable access to quality care.

A third major strategy to facilitate equitable access to and delivery of MNCH services is to strengthen and scale-up health system capacity and delivery of essential interventions closer to the communities being served [19, 52, 58, 68–71], which is exemplified by the rapid and notable impact achieved through Kenya’s Community Health Strategy. In contrast to the KEPH policy, study findings indicated that Kenya’s Community Health Strategy has had a more rapid and notable impact to date. The use of CHWs to promote integrated MNCH services and community-based educational campaigns, and increased provision of services through mobile clinics and primary care dispensaries likely facilitated Kenya’s attainment of high coverage levels of immunizations, malaria prevention, and PMTCT services. Other studies in Kenya [5, 12, 72] and other resource-limited countries [73–76] have also demonstrated increased availability, awareness and utilization of MNCH services, improved health behaviors, and improved health outcomes in areas where community health services have been implemented and/or expanded [5, 12, 72]. Despite the initial success of Kenya’s Community Health Strategy, at the end of our study period only a few community units were fully functional and others were still dependent on donor aid. Thus, as Kenya continues to accelerate its efforts to reduce child mortality, these concerns will need to be addressed with sustainable measures to increase MOH support for CHWs and to ensure the effective delivery of the community KEPH package.

This study provides one of the first country-case studies assessing challenges and facilitators to achieving MDG#4 from a country that made some, but insufficient, progress towards the goal. By bringing together diverse sources of data, including national indicator data, country-authored health policies and strategies and qualitative data from key informants and community women, the case study was able to assess national-level efforts intended to reduce under-five mortality and the degree of implementation and impact. Nevertheless, there are limitations for each of the study components. For the review of national MNCH policies and strategies, many documents were published towards the end of the study period (e.g., after 2007) as the country was heightening its focus on MDG#4. While these documents contained a retrospective assessment of the earlier part of the study period, assessments of the impact of more recent policies or strategies were not yet available. Moreover, country policies and strategies covered different and sometimes overlapping time periods, making it difficult to distinguish current from outdated information, and whether a stated plan had been implemented unless specifically stated in a document. Input from co-authors affiliated with the WHO and the Ministry of Health helped to clarify some confusion.

The interviews and focus groups were limited to a non-random sample of participants and conducted primarily in Nairobi and Embu which may not fully reflect the opinions of those from other areas of Kenya. Specifically, we acknowledge that health facility births were overrepresented in our focus group participants (both urban and rural). However, study sites were chosen from areas that reflected national trends in under-five mortality in Kenya and participants were selected to provide representation across a spectrum of MNCH experiences including some key informants with national-level responsibilities and community women from both urban and rural areas. Another limitation is that participants more often recalled their recent experiences and opinions on MNCH even though we asked them to reflect on long-term changes. Finally, language fluency may have affected comprehension of the interviews and focus group discussion. Although pilot testing revealed no substantial comprehension problems, some key informants were uncomfortable conducting the interviews in English, while some focus group discussions included women with variable levels of Swahili fluency.

This comprehensive case study provides information on the implementation of policies and strategies Kenya has established to address inequities in MNCH intervention delivery, coverage, utilization, and access. We have noted ways in which policies and strategies are being successfully implemented as well as gaps in implementation that may, in part, account for Kenya’s insufficient progress towards MDG#4. In order to further reduce under-five mortality, Kenya must continue to address cost barriers related to seeking MNCH services and to improve health system infrastructure, personnel, and essential medicines to cover more underserved areas and meet increasing demands, particularly at the primary care level, as outlined in the KEPH. To maintain the gains made through community outreach and service delivery, community units need to be linked with facilities and more fully supported by the government, rather than external donors. Additional CHWs should be trained and provided with incentives to ensure motivation and retention. These recommendations can also be applied to other countries seeking to improve child survival and MNCH care through similar policies and strategies. Kenya’s experiences can inform other countries seeking to implement similar approaches to reduce inequities and child mortality in their post-2015 plans.

## Supporting information

S1 Table Kenya policy, strategy, and other national documents reviewed. (DOCX)
