# Supplementary material for: The introduction of new policies and strategies to reduce inequities and improve child health in Kenya: A country case study on progress in child survival, 2000-2013
Source: PLoS One. 2017 Aug 1;12(8):e0181777. doi: 10.1371/journal.pone.0181777 (PMC5538680; doi:10.1371/journal.pone.0181777)
Supplement: S1 Table — (DOCX) [file pone.0181777.s001.docx]

# SUPPLEMENTAL TABLE

**Table S1. Kenya policy, strategy, and other national documents reviewed**

| **Document title (dates if not otherwise specified in title)** |
| --- |
| ***In-depth* (Newest to oldest)*** |
| Transforming Health: Accelerating Attainment of Health Goals. Kenya Health Sector Strategic and Investment Plan. The Second Medium Term Plan for Health (2013, DRAFT) |
| Maternal, Newborn and Child Health Bill (2013, DRAFT) |
| Health Sector Function Assignment and Transfer Policy Paper (2013) |
| Kenya Health Policy, 2012-2030 |
| Constitution of Kenya (2010) |
| Kenya Essential Medicines List (2010) |
| Access to Essential Medicines in Kenya – Health facility survey (2009) |
| Access to Essential Medicines in Kenya – Household survey (2009) |
| Child Survival and Development Strategy, 2008-2015 |
| Second National Health Sector Strategic Plan Mid Term Review (2007) |
| Kenya Vision 2030 (2007) |
| ***Brief review† (Newest to oldest)*** |
| Guidelines for Prevention of Mother to Child Transmission of HIV/AIDS in Kenya (2012) |
| Food and Nutrition Action Plan (2012) |
| Proceedings from National Stakeholder’s Workshop on Acceleration of Maternal, Newborn and Child Survival (2011) |
| Kenya IMCI Health Facility Survey (2010) |
| Health Sector Strategic Plan for Health Information System (2009) |
| Reversing the Trends, The Second National Health Sector Strategic Plan: Clinical Management and Referral Guidelines, Vols. I, II & III (2009):   - Volume I: Clinical Guidelines for Management and Referral of Common Conditions at Level 1: The Community - Volume II: Clinical Guidelines for Management and Referral of Common Conditions at Level 2-3: Primary Care - Volume III: Clinical Guidelines for Management and Referral of Common Conditions at Levels 4–6: Hospitals |
| Republic of Kenya Health Sector Report for the Assessment of the Health Information System of Kenya (2008) |
| National Food Security and Nutrition Policy (2008) |

*Primary documents extensively reviewed to obtain information on each content area identified in the abstraction guide

†Documents reviewed to obtain additional information on specific content areas not sufficiently covered by the primary documents
